# Advancing EGFR mutation subtypes prediction in NSCLC by combining 3D pretrained ConvNeXt, radiomics, and clinical features

**DOI:** 10.3389/fonc.2024.1464555

**Published:** 2024-11-15

**Authors:** Peng Hao, Yinghong Yu, Chan-Tao Huang, Fang Zhou, Yi-Kai Xu, Jiancheng Yang, Jun Xu

**Affiliations:** ^1^ Department of Diagnostic Imaging Center, Nanfang Hospital, Southern Medical University, Guangzhou, Guangdong, China; ^2^ AIgorithm Department, Dianei Technology, Shanghai, China; ^3^ Computer Vision Laboratory, Swiss Federal Institute of Technology Lausanne (EPFL), Lausanne, Switzerland; ^4^ Department of Hematology, Nanfang Hospital, Southern Medical University, Guangzhou, Guangdong, China

**Keywords:** NSCLC, EGFR, CT, deep learning, radiomic

## Abstract

**Purpose:**

The aim of this study was to develop a novel approach for predicting the expression status of Epidermal Growth Factor Receptor (EGFR) and its subtypes in patients with Non-Small Cell Lung Cancer (NSCLC) using a Three-Dimensional Convolutional Neural Network (3D-CNN) ConvNeXt, radiomics features and clinical features.

**Materials and methods:**

A total of 732 NSCLC patients with available CT imaging and EGFR expression data were included in this retrospective study. The region of interest (ROI) was manually segmented, and clinicopathological features were collected. Radiomic and deep learning features were extracted. The instances were randomly divided into training, validation, and test sets. Feature selection was performed, and XGBoost was used to create solo models and combined models to predict the presence of EGFR and subtypes mutations. The effectiveness of the models was assessed using ROC and PRC curves.

**Results:**

We established the following models: Model_CNN_, Model_radiomic_, Model_clinical_, Model_CNN+radiomic_, Model_CNN+clinical_, Model_radiomic+clinical_, and Model_CNN+radiomic+clinical_, which were based on deep learning features, radiomic features, clinical data and combinations of these, respectively. In predicting EGFR mutations, Model_CNN+radiomic+clinical_ demonstrated superior performance compared to other prediction models, achieving an AUC of 0.801. For distinguishing between EGFR subtypes ex19del and L858R, Model_CNN+radiomic_ reached the highest AUC value of 0.775.

**Conclusions:**

Both deep learning models and radiomic signature-based models offer reasonably accurate non-invasive predictions of EGFR status and its subtypes. Fusion models hold the potential to enhance noninvasive methods for predicting EGFR mutations and subtypes, presenting a more reliable prediction approach.

## Introduction

Lung cancer stands as the most lethal form of cancer globally, presenting the highest mortality rate among all malignancies. Approximately 80% of lung cancers belong to the histological category of non-small-cell lung cancer (NSCLC) (1). Currently, clinical treatment for lung cancer focuses on controlling local lesions and metastases. Targeted therapy offers advantages such as precise targeting, minimal side effects, ease of use, and high therapeutic efficacy (2).

One of the key proteins involved in lung cancer is the epidermal growth factor receptor (EGFR). Lung cancer can be classified into two categories: EGFR mutation-positive tumors and non-mutated tumors (EGFR wild type) (3). The EGFR ex19 Del and L858R mutations account for 90% of EGFR mutation-positive cases and affect approximately 50% of individuals with lung adenocarcinoma in the Asian population. Patients with wild-type EGFR cannot benefit from EGFR-tyrosine kinase inhibitor (TKI) treatment (4). Studies have shown that patients with EGFR ex19del mutation have better prognosis and treatment response compared to those with L858R mutation. For instance, in the context of osimertinib combination therapy or osimertinib targeted therapy alone, patients with EGFR ex19del mutation have shown longer progression-free survival (PFS) compared to those with L858R mutation (5). Therefore, accurately defining the EGFR mutation subgroups can be crucial in ensuring precise diagnosis and individualized treatment for NSCLC patients. The accuracy of EGFR gene assessment using biopsy samples may be compromised due to significant intratumor heterogeneity. Additionally, some patients may have inoperable lung adenocarcinoma or may not be able to undergo biopsy due to factors such as endurance, willingness, or cost. Therefore, a non-invasive approach to determine EGFR mutation status and subtypes is needed. Computer tomography (CT) is commonly used for lung cancer diagnosis. Machine learning (ML) and artificial intelligence can thoroughly evaluate tumors, improve the sensitivity and specificity of diagnostic imaging, and provide a non-invasive method for lung cancer-related diagnosis (6, 7). However, the aforementioned deep learning (DL) study only focused on identifying the presence of EGFR mutations (wild-type versus ex19Del+L858R), without specifically differentiating between the subtypes of EGFR mutations (ex19Del vs L858R), or only using machine learning (8–10).

In this study, we aimed to directly distinguish between EGFR (+) and EGFR (-) and then differentiate between two common subtypes of EGFR mutations, ex19Del and L858R, using DL and ML analysis of primary lung adenocarcinoma. The findings of this study may contribute to a more comprehensive and non-invasive discrimination of EGFR mutations and subtypes. This, in turn, could serve as a foundation for developing individually tailored and effective diagnosis and treatment plans for lung cancer patients.

## Materials and methods

### Patients inclusion

From May 2012 to August 2021, a retrospective study was conducted on all CT scans of non-small cell lung cancer (NSCLC) patients from the Picture Archiving and Communication System (PACS) at Nanfang Hospital. A total of 1080 patients with pathologically proven lung cancer who underwent surgery or received biopsy were included in this study. The clinical features of the patients were retrieved from the hospital information system. Inclusion criteria for this study were: (1) patients with confirmed EGFR gene mutation status and pathological testing of tumor specimens; (2) patients with pretreatment CT images; (3) patients with complete clinical data (including sex, age, smoking, T stage, and lesion size). Exclusion criteria were: (1) patients who received treatment before CT scan; (2) patients with a time interval longer than one month between CT examination and treatment; (3) patients with multiple tumor nodules in the lung; and (4) patients with tumor lesions near the hilar that could not be separated from neighboring hilar architecture. Based on these criteria, a total of 732 patients were included in the study. The TNM system based on the American Joint Committee on Cancer (AJCC) manual was used for staging (11).

In this study, a total of 1080 cases were initially included. However, 348 cases were excluded for various reasons. These exclusions included cases without pre-treatment CT images (n=132), cases with multifocal primary tumors (n=70), cases where the time interval between biopsy or surgery was more than 12 weeks (n=50), cases with tumors in the mediastinum (n=10), cases with severe infection (n=10), and cases with mutations in exons 18 and 20 (n=16). The latter exclusion was due to the insufficient number of tumors with these specific mutations for reasonable statistical analysis.

The focus of this study was on mutations in exons 19del and L858R of the EGFR gene. After the exclusions, the final study cohort consisted of 732 patients. Among these patients, there were a total of 351 cases with EGFR mutations, with 195 cases of EGFR ex19del and 156 cases of EGFR L858R. This distribution represents approximately 55% of cases with EGFR ex19del and 45% of cases with EGFR L858R. For more detailed information on the distribution of cases and mutations, please refer to **Tables 1**, **2**.

**Table 1:** Distribution of data for predicting EGFR mutations.

	Total	EGFR+	EGFR-
Total	732	351	381
Training group	512	246	266
Validation group	73	35	38
Testing group	147	70	77

EGFR (+), EGFR mutation-positive; EGFR (-), EGFR mutation-negative.

**Table 2:** Distribution of data for distinguishing EGFR ex19del from L858R.

	Total	EGFR ex19del	EGFR L858R
Total	351	195	156
Training group	245	136	109
Validation group	35	19	16
Testing group	71	40	31

Among the patients included in the study, 351 out of 732 (48%) tested positive for an EGFR mutation, while 381 out of 732 (52%) tested negative for an EGFR mutation. We observed a significant association between EGFR mutations and non-smoking female patients with non-small cell lung cancer, as shown in **Supplementary Table S1**.

Out of the total cases with EGFR mutations, 195 (55%) were identified as EGFR Ex19del and 156 (45%) were identified as EGFR L858R. This distribution indicates that EGFR Ex19del is slightly more prevalent than L858R. Additionally, we found that the L858R mutation was associated with older patients, as indicated in **Supplementary Table S2**.

### CT scanning

The patients were examined using either a 256-slice iCT scanner (Philips Healthcare, Best, Netherlands) or Siemens Medical Solutions’ Sensation 64 or Definition AS scanner (Forchheim, Germany). The scanning parameters for the two scanners were as follows: tube rotation time of 0.5 s, pitch of 0.87 or 1.2, detector collimation of 128 x 0.625 or 64, tube voltage of 120 kV, tube current of 100-300 mA, field view of 350 mm, matrix of 512x512, slice thickness of 1-5 mm, reconstruction interval of 1 mm.

### Histopathology and EGFR status determination

The histopathological type of non-small cell lung cancer was determined by diagnostic pathologists using the 2011 International and Multidisciplinary Classification and the criteria put forward by the World Health Organization (WHO) 2015 guidelines for lung cancer categorization and the International Association for the Study of Lung Cancer/American Thoracic Society/European Respiratory Society. The EGFR mutation status was determined using a real-time fluorescent PCR-based amplification refractory mutation system and a human EGFR gene mutation real-time reverse transcription-polymerase chain reaction diagnostic kit (AmoyDx, Xiamen, China). The mutation status of EGFR exons 18, 19, 20, and 21 was analyzed.

### Clinical information

We extracted five features from the clinical information, including sex, age, smoking, T stage, and lesion size. The clinical features of the patients were retrieved from the hospital information system.

### Radiomic analysis

We extracted 1051 radiomic features from the image ROI and corresponding ROI mask. We then used Boruta (12) for feature selection on the training dataset. Boruta operates on two principles: shadow features and biological distribution. This algorithm autonomously conducts feature selection on the dataset.

To examine the differences in radiomics features between the EGFR mutation-positive EGFR (+) and EGFR mutation-negative EGFR (-) groups, we conducted feature selection with Boruta. The algorithm identified 11 radiomics. These selected features can potentially serve as predictive markers for EGFR mutations. Further analysis and validation are needed to confirm their significance and utility in clinical practice. Additionally, for the specific EGFR mutation subtypes (EGFR ex19del and L858R), we performed radiomics feature extraction and identified 9 radiomics (details in **Supplementary Methods**).

### Model for deep learning

**Figure 1:**
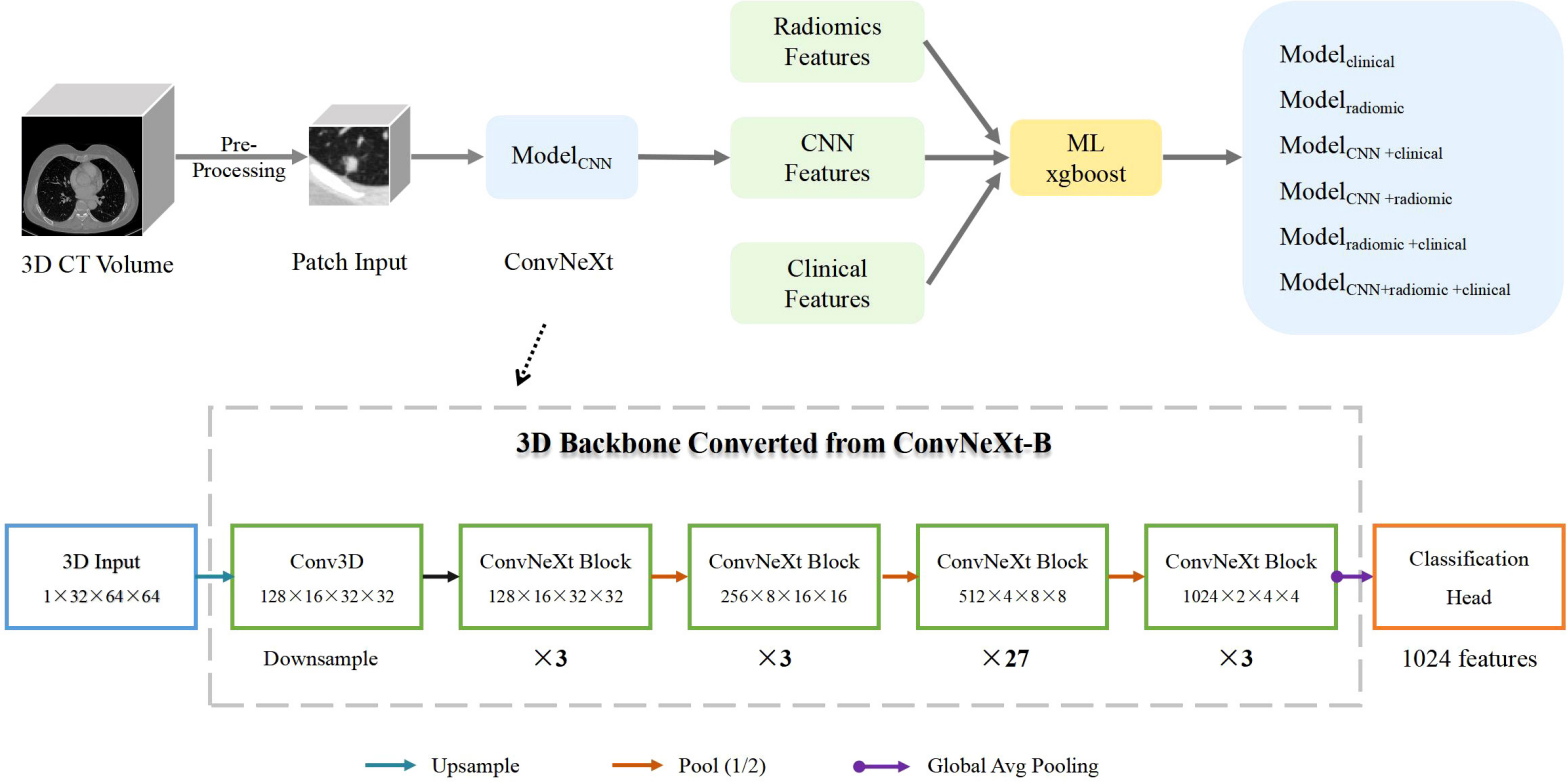
Pipeline Overview. Model_CNN_ is a 3D ImageNet-22K pre-trained model based on ConvNeXt-B. The conversion from 2D to 3D is enabled by the ACS convolution technique. Model_clinical_ is a machine-learning xgboost model trained on clinical information. Model_radiomic_ is an xgboost model trained on radiomcs features. Model_CNN+clinical_ combines ConvNeXt model predictions with clinical information. Model_radiomic+clinical_ combines radiomics features with clinical information. Model_CNN + radiomic+clinical_ incorporates ConvNeXt model predictions, radiomics features, and clinical information. The structure of the ConvNeXt-B model is shown in the lower half of the figure.

Our research focuses on developing a deep learning framework for accurately predicting gene mutations in nodules. To achieve this, we utilized ConvNeXt, a powerful deep learning model that achieved top-1 accuracy on the ImageNet dataset in early 2022 (13). ConvNeXt is composed of standard convolutional modules and has demonstrated exceptional accuracy and scalability. For our experiments, we specifically used the ConvNeXt-B model, which consists of 89 million parameters (14, 15), the pipeline overview in shown in **Figure 1**. Acknowledging the three-dimensional nature of CT images, we utilized ACS conv (https://github.com/M3DV/ACSConv) to convert a 2D pre-trained model based on ImageNet-22K into a 3D model (16). In our approach, we preprocessed the input images by cropping them around the nodule center with a size of 32×64×64 (64×64 pixels in the axial plane, 32 frames). We then upsampled the images by a factor of 2 to 64×128×128 before feeding them into the model. Features are extracted through downsampling until the size of the feature map becomes 2×4×4. Finally, we applied global average pooling to generate a 1024-dimensional feature vector for classification. For the gene mutation classification task, we employed a simple Multi-Layer Perceptron (MLP) with one hidden layer. This MLP takes the 1024-dimensional feature vector as input and performs the final classification. During our experiments, we randomly selected data for training, validation, and testing, with a ratio of 7:1:2. The validation dataset was used to select the best model, and we reported the test results on both the validation and test sets. The deep learning model was implemented with Python 3.8.12 and PyTorch 1.11.0.

### Feature fusion

In our research, we investigated four distinct strategies for feature integration. Each fusion method employed the use of XGBoost to construct the model (17). The initial fusion, termed as Model_CNN+clinical_, integrated deep learning features (specifically the predictive probability of ConvNeXt) with clinical data (sex, age, smoking history, T stage, and lesion size). The second fusion, identified as Model_CNN+radiomic_, merged deep learning features with radiomic characteristics. The third fusion, labeled as Model_radiomic+clinical_, combined radiomic attributes with clinical information. Lastly, the fourth fusion model, referred to as Model_CNN+radiomic+clinical_, amalgamated deep learning features, radiomics attributes, and clinical data. To evaluate the performance of these feature fusion approaches, we utilized ROC (Receiver Operating Characteristic) and PRC (Precision-Recall Curve) curves. These curves provide valuable insights into the model’s ability to discriminate between positive and negative cases, as well as its precision and recall. Accuracy, Recall, Precision, Specificity, and F1-score are calculated using the Youden index, which is defined as sensitivity + specificity – 1 (18).

### Statistical analysis

Statistical analysis was performed using IBM SPSS Statistics version 25.0. Continuous variables were analyzed using the two independent samples t-test or Mann-Whitney U test, depending on the distribution of the data. Categorical variables were analyzed using the chi-square test or Fisher’s exact test. The significance of the ML model’s performance in differentiating between EGFR+ and EGFR- groups, as well as between ex19del and L858R mutations, was assessed using the same statistical methodologies.

## Results

### Performance in predicting EGFR mutation

**Figure 2:**
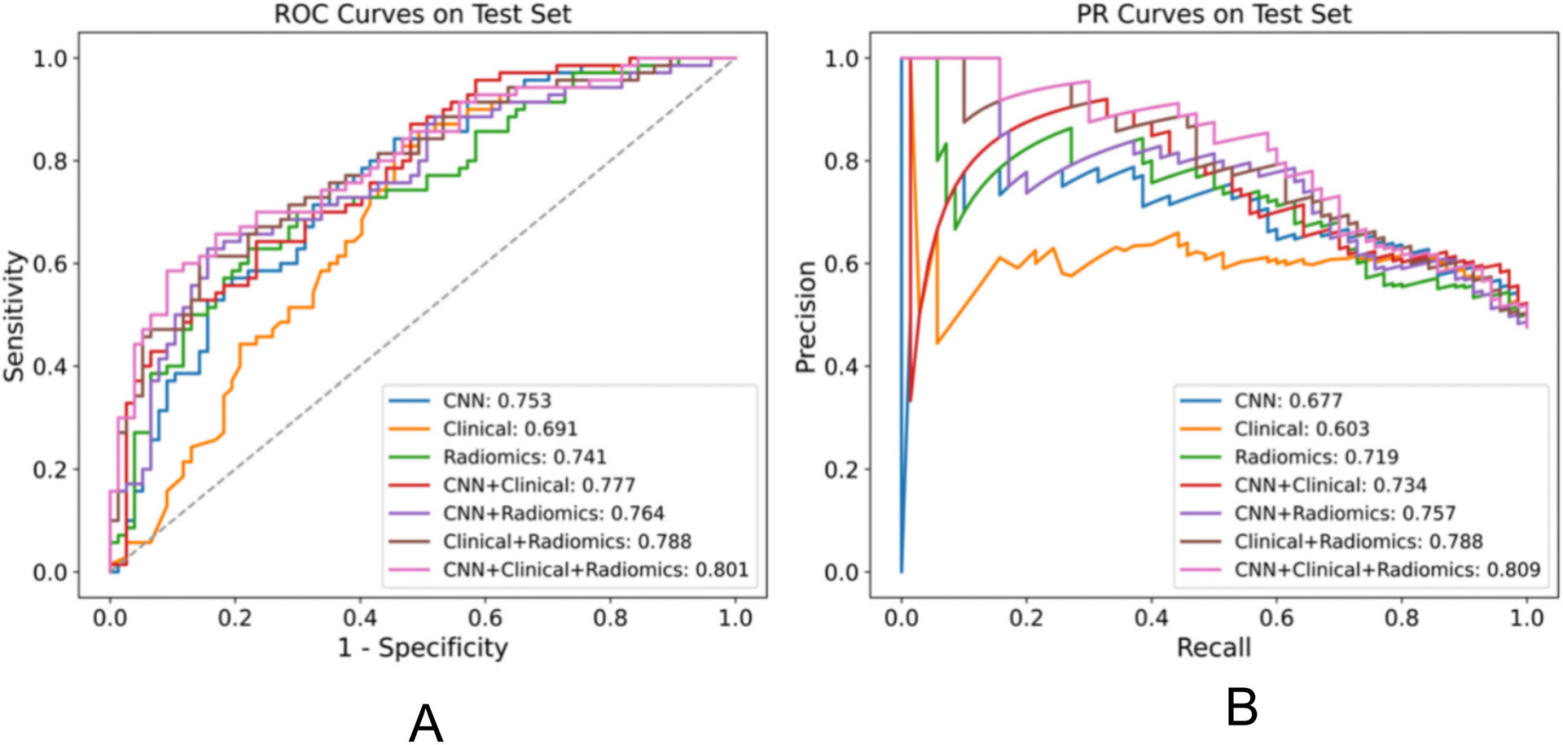
Performance comparison of various models for the EGFR mutation task on the test set, displaying the AUC for each model in the legend. **(A)** ROC plot **(B)** PR plot.

We evaluated the performance of different models in predicting EGFR mutation status using the area under the curve (AUC) metric as presented in **Table 3**. In **Table 3**, pairwise DeLong tests were conducted between the first three columns (CNN, clinical, and radiomic models) and the last four columns (fusion models), yielding p<0.05. This indicates a significant difference in AUC between the multimodality fusion models and the single modality models. However, there were no significant differences between the single modality models (p>0.05), nor between the multimodality models themselves (p>0.05). In addition, **Table 4** displays supplementary performance metrics (including accuracy, recall, precision, specificity, and F1-score), while **Figure 2** showcases the ROC and PRC curves. For the CNN probability prediction model, the AUC values were 0.73, 0.78, and 0.753 in the training, validation, and test groups, respectively. These results indicate that the CNN model has moderate predictive ability for EGFR mutation. To further improve the predictive performance, we developed a fusion model that combines deep learning, radiomics features, and clinical information. This model, called Model_CNN + radiomic + clinical_, achieved higher AUC values compared to the CNN model. Specifically, the AUC values for the fusion model were 0.81, 0.848, and 0.801 in the training, validation, and test groups, respectively. These results demonstrate that integrating multiple data sources can enhance the accuracy of EGFR mutation prediction. Overall, the fusion model shows promising performance in predicting EGFR mutation status and may have potential clinical utility in guiding treatment decisions for non-small cell lung cancer patients.

**Table 3:** AUC performance of different models for predicting EGFR mutations across the training, validation, and test sets.

AUC	Model _CNN_	Model _clinical_	Model _radiomic_	Model _CNN+clinical_	Model _CNN+radiomic_	Model _radiomic+clinical_	Model _CNN+radiomic+clinical_
Training	0.73	0.687	0.714	0.785	0.788	0.802	**0.81**
Validation	0.78	0.761	0.774	0.863	0.823	0.842	**0.848**
Testing	0.753	0.691	0.741	0.777	0.764	0.788	**0.801**

The best-performing value in each column is highlight in bold.

**Table 4:** Additional performance metrics of different models for predicting EGFR mutations in the testing set.

	AUC	Accuracy	Recall	Precision	Specificity	F1-score
Model_CNN_	0.753	**0.694**	0.743	**0.658**	0.649	**0.698**
Model_clinical_	0.691	0.646	0.757	0.602	0.545	0.671
Model_radiomic_	0.741	0.66	0.729	0.622	0.597	0.671
Model_CNN+clinical_	0.777	0.667	**0.786**	0.618	0.558	0.692
Model_CNN+radiomic_	0.764	0.68	0.7	0.653	**0.662**	0.676
Model_radiomic+clinical_	0.788	0.68	0.771	0.635	0.597	0.697
Model_CNN+radiomic+clinical_	**0.801**	0.68	0.757	0.639	0.61	0.693

The best-performing value in each column is highlight in bold.

### Performance in distinguishing EGFR Ex19del and L858R mutations

**Figure 3:**
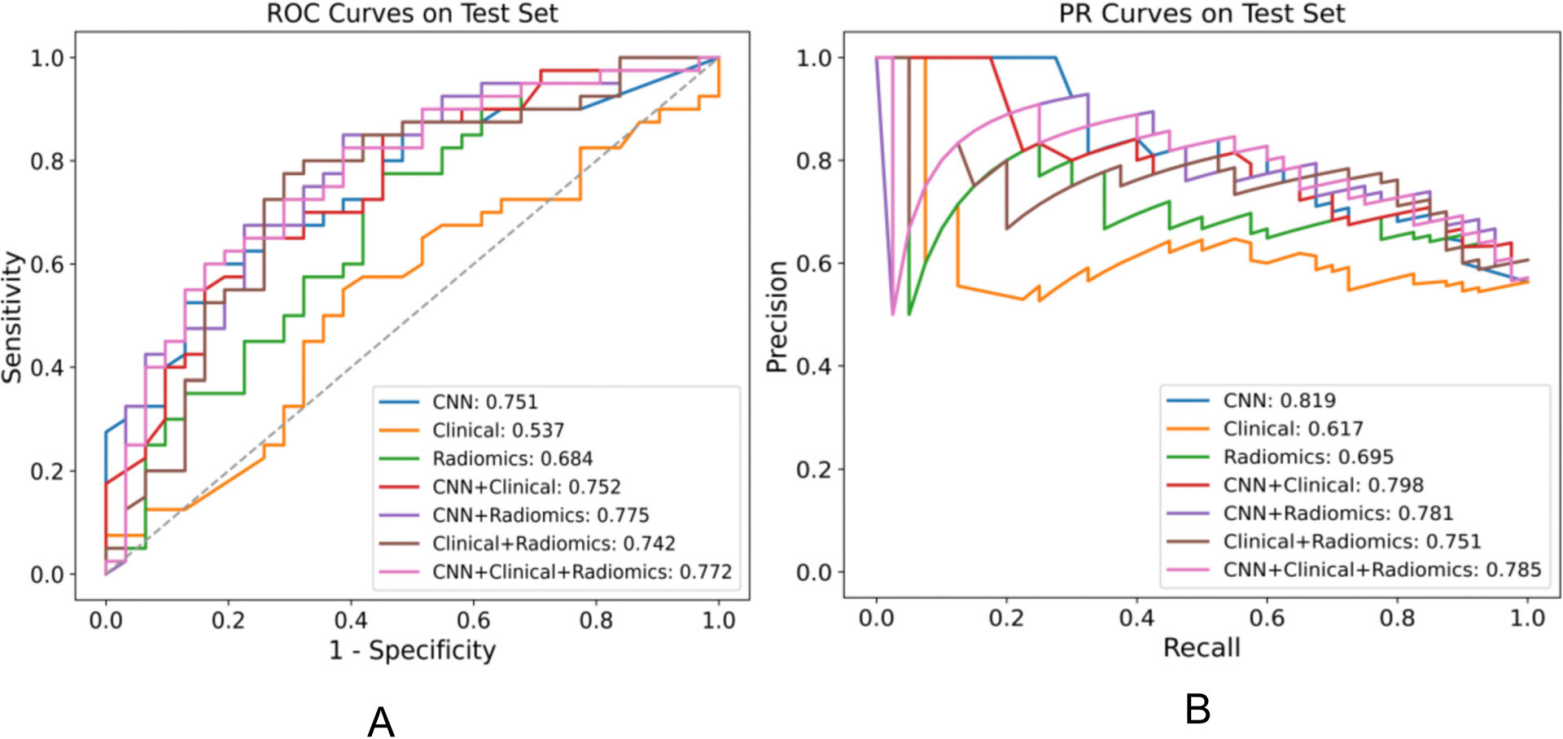
Performance comparison of various models for distinguishing EGFR ex19del, L858R on the test set, displaying the AUC for each model in the legend. **(A)** ROC plot **(B)** PR plot.

We assessed various models’ efficacy in distinguishing between EGFR Ex19del and L858R mutations, with AUC detailed in **Table 5**. Additionally, **Table 6** presents supplementary performance metrics, and **Figure 3** illustrates the ROC and PRC curves. For the deep learning model, the AUC values were 0.781, 0.765, and 0.751 in the training, validation, and test groups, respectively. These results indicate that the deep learning model has moderate predictive ability for distinguishing between these two mutation types. To further improve the performance, we developed a fusion model called Model_CNN+radiomic_. This model combines deep learning with radiomics features and has shown improved predictive performance. Specifically, the fusion model achieved AUC values of 0.811 in the validation group and 0.775 in the test group. These results suggest that the fusion model is better at distinguishing between EGFR Ex19del and L858R mutations compared to the deep learning model alone. Overall, our findings demonstrate that the fusion model, combining deep learning and radiomics features, has superior performance in accurately distinguishing between EGFR Ex19del and L858R mutations.

**Table 5:** AUC performance of different models for distinguishing ex19del, L858R across the training, validation, and test sets.

AUC	Model _CNN_	Model _clinical_	Model _radiomic_	Model _CNN+clinical_	Model _CNN+radiomic_	Model _radiomic+clinical_	Model _CNN+radiomic+clinical_
Training	0.781	0.613	0.733	0.767	0.775	0.726	**0.793**
Validation	0.765	0.589	0.724	0.771	**0.811**	0.763	0.809
Testing	0.751	0.537	0.684	0.752	**0.775**	0.742	0.772

The best-performing value in each column is highlight in bold.

**Table 6:** Additional performance metrics of different models on distinguishing ex19del and L858R in the testing set.

	AUC	Accuracy	Recall	Precision	Specificity	F1-score
Model_CNN_	0.751	**0.676**	**0.65**	0.743	0.71	**0.693**
Model_clinical_	0.537	0.535	0.425	0.63	0.677	0.507
Model_radiomic_	0.684	0.62	**0.65**	0.667	0.581	0.658
Model_CNN+clinical_	0.752	0.62	0.4	0.842	0.903	0.542
Model_CNN+radiomic_	**0.775**	0.648	0.525	0.778	0.806	0.627
Model_radiomic+clinical_	0.742	0.649	0.55	0.759	0.774	0.638
Model_CNN+radiomic+clinical_	0.772	0.577	0.3	**0.857**	**0.935**	0.444

The best-performing value in each column is highlight in bold.

### Analysis of feature importance and cluster maps

**Figure 4:**
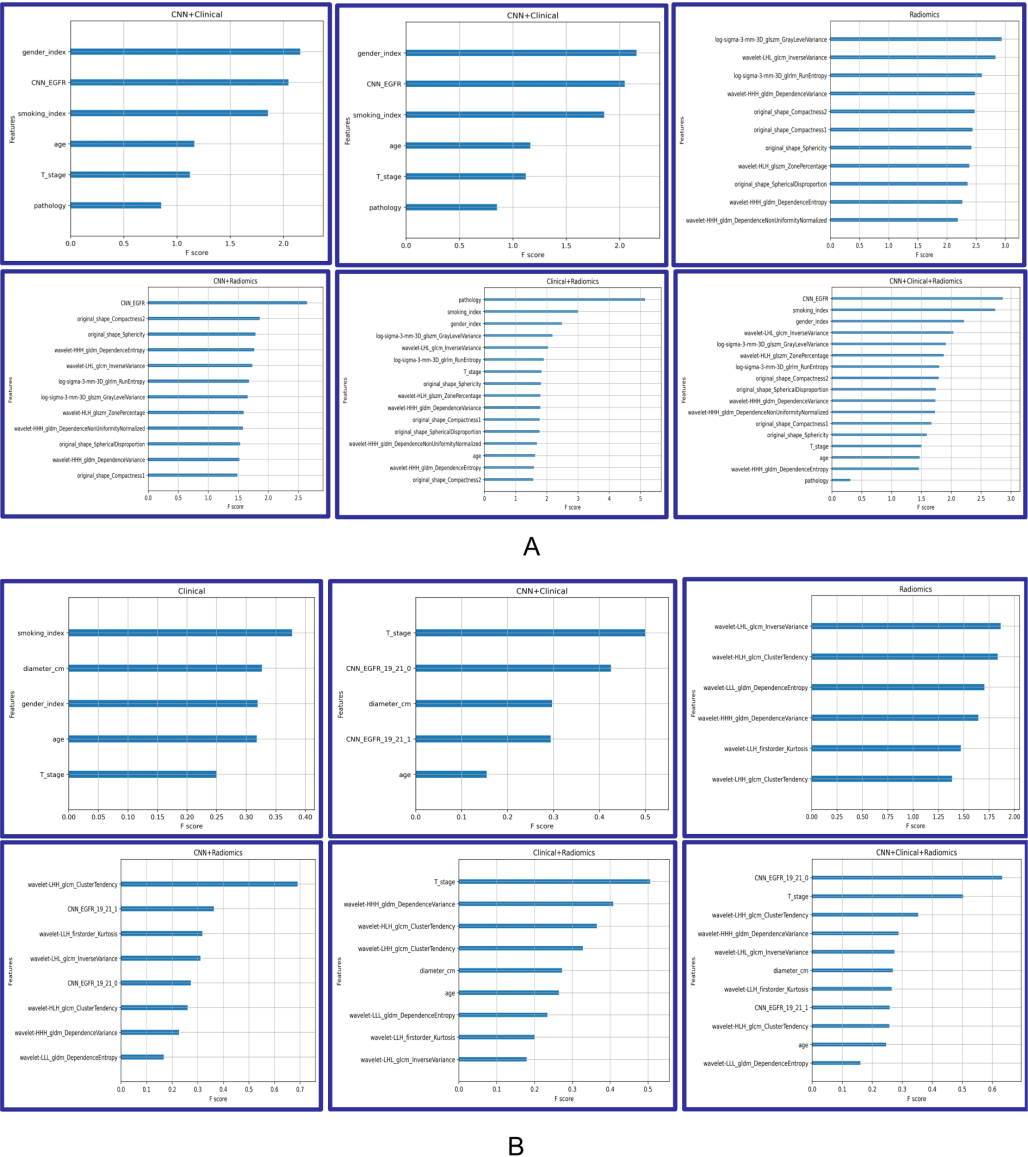
Feature importance of Model_clinical_, Model_radiomic_, Model_CNN+clinical_, Model_CNN+radiomic_, Model_radiomic+clinical_, and Model_CNN+radiomic+clinical_. **(A)** Predicting EGFR mutations. **(B)** Distinguishing ex19del and L858R.

The analysis of feature importance in the fusion model provides us with valuable insights, as depicted in **Figure 4**. When discriminating EGFR mutations, in the CNN + radiomic + clinical fusion model, the most important features are the CNN extracted features, smoking index, and gender, followed by radiomic features. For discriminating EGFR Ex19del and L858R mutations, CNN and nodule size, along with radiomic features, are comparatively more significant.

**Figure 5:**
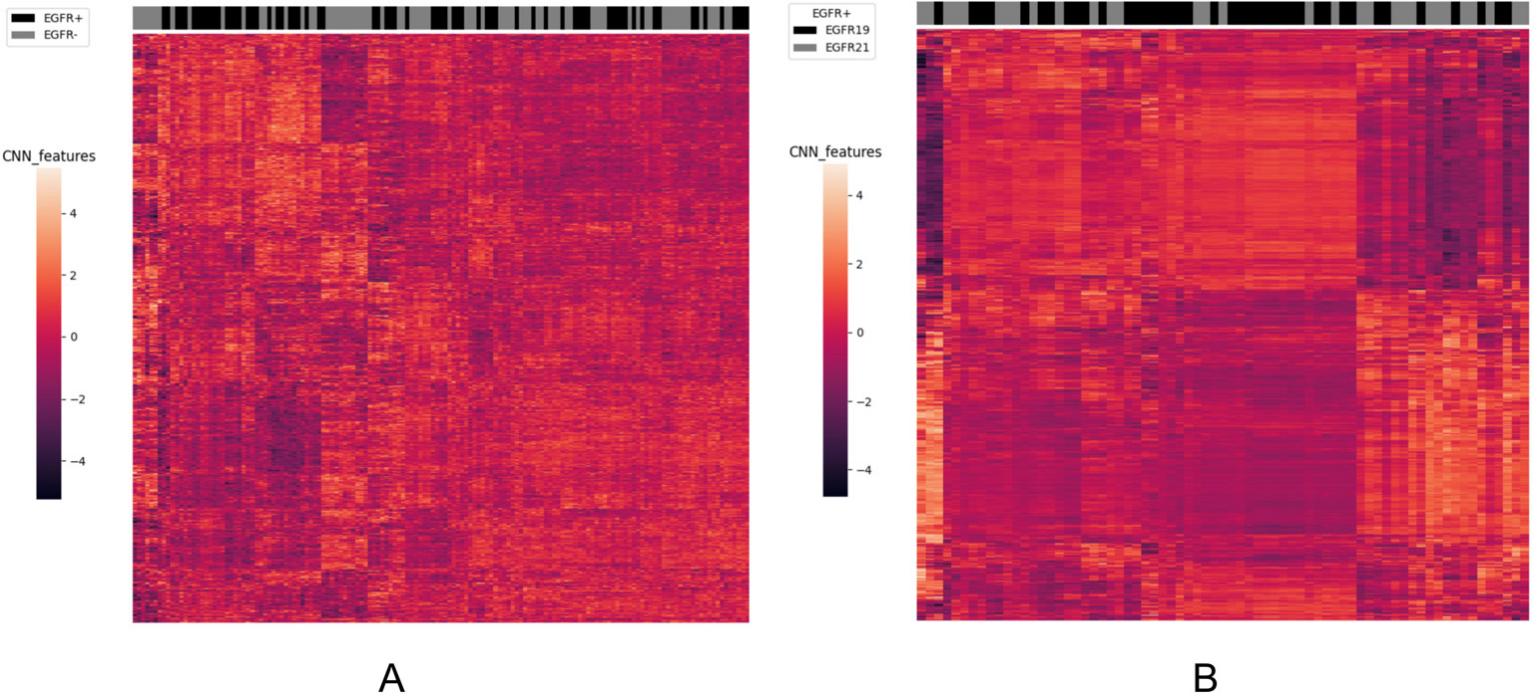
The clustering relationship of ConvNeXt features extracted by the model. **(A)** In the task of predicting EGFR mutations, the x-axis represents 147 nodules from the test set, and the y-axis represents the 1024-dimensional features extracted by CNN. Each feature has been normalized. Nodules within the same cluster (adjacent columns) exhibit similar radiomics characteristics in Euclidean space. The black gray bar indicates the semantic tag EGFR +/- for each nodule. **(B)** In the task of distinguishing ex19del and L858R, the x-axis represents 71 nodules from the test set, and the y-axis represents the 1024-dimensional features extracted by CNN. Again, each feature has been normalized. Nodules within the same cluster exhibit similar radiomics characteristics, and the black gray bar indicates the semantic label EGFR ex19del/EGFRL858R for each nodule.

**Figure 6:**
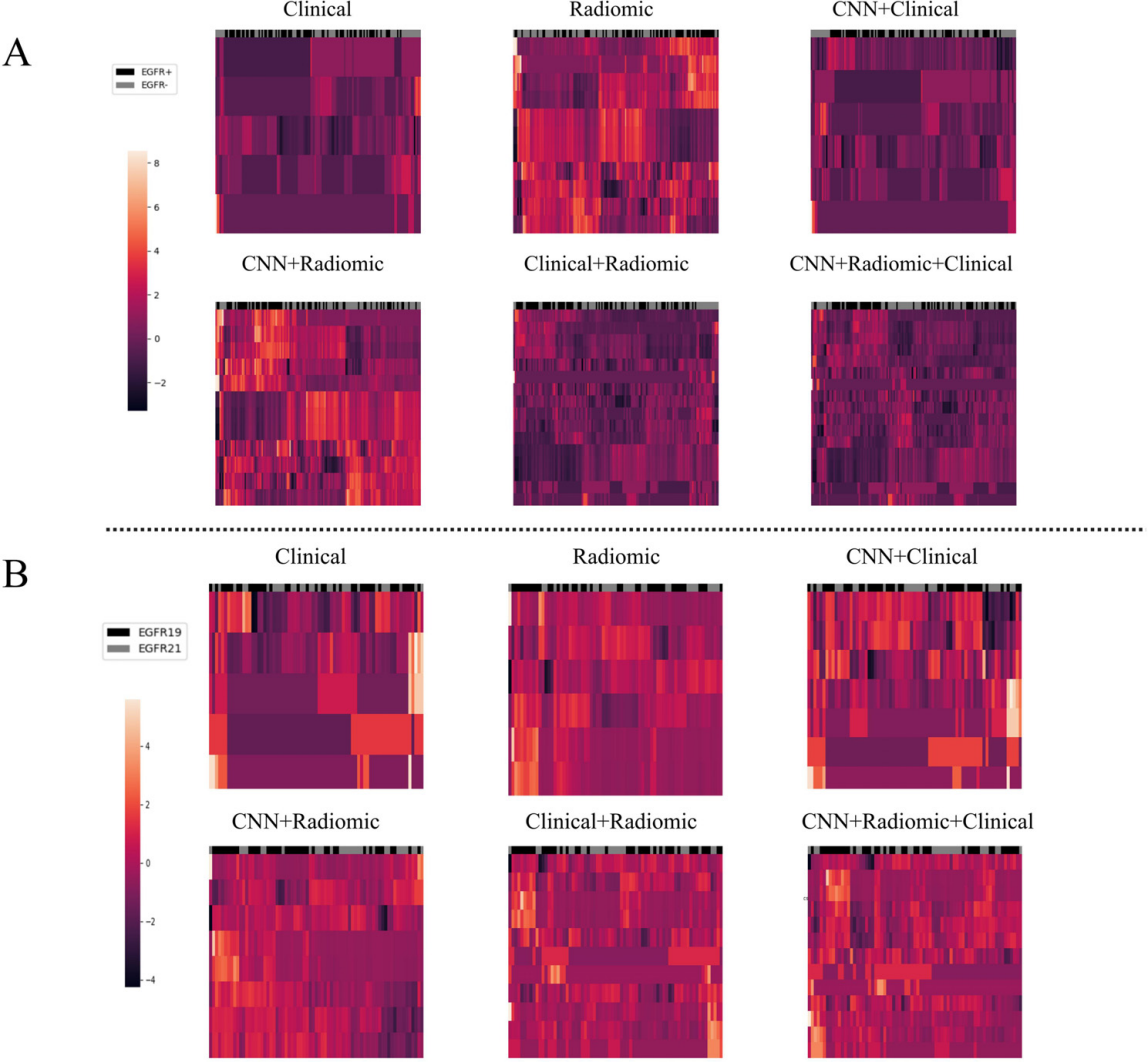
The clustering relationship of different models based on various features. **(A)** In the task of predicting EGFR mutations, the x-axis represents 147 nodules from the test set, and the y-axis represents the features of different models such as Model_clinical_, Model_radiomic_, Model_CNN+clinical_, Model_CNN+radiomic_, Model_radiomic+clinical_, and Model_CNN+radiomic+clinical_. Each feature has been normalized. Nodules in the same cluster (adjacent columns) have similar radiomic characteristics in Euclidean space. The black gray bar indicates the semantic tag EGFR +/- for each nodule. **(B)** In the task of distinguishing ex19del and L858R, the x-axis represents 71 nodules from the test set, and the y-axis represents the features of the different models. Again, each feature has been normalized. Nodules in the same cluster have similar radiomic characteristics, and the black gray bar indicates the semantic label EGFR ex19del/EGFRL858R for each nodule.

To analyze the performance of the CNN-extracted features, we examined the clustering relationship between the 1024 CNN features extracted by the model prior to classification and the labels in **Figure 5**. It can be observed that in both classification tasks, the unsupervised clusters of the 1024 deep-learned radiomics features extracted from ConvNext align closely with the semantic labels. In other words, the continuous regions on the black-grey bars share numerous similar features, respectively. Similarly, in **Figure 6**, across both tasks, we also observed a rather good clustering relationship between the fusion of CNN features, clinical features, radiomics features, and the labels. This suggests that the features we extracted possess a certain discriminatory ability and exhibit improved diagnostic performance after fusion.

## Discussion

In this study, we aimed to develop a fusion model that combines clinical, radiomic, and deep learning data to predict EGFR mutation subtypes in non-small cell lung cancer (NSCLC) patients. Compared to models based solely on radiomic or deep learning features, our fusion model (Model_CNN+radiomic+clinical_) demonstrated superior effectiveness. Previous studies have primarily focused on using deep learning approaches to predict the overall EGFR mutation status without clearly distinguishing between different mutation subtypes (6, 19). Zhao et al. developed a deep learning system based on 3D CNNs to automatically predict EGFR mutant pulmonary adenocarcinoma in CT images, with AUCs of 75.8% and 75.0% for holdout test set and public test set, respectively (20). However, the analysis did not cover EGFR mutation subtypes. In earlier investigations, Liu et al. only employed radiomics characteristics predicted the overall EGFR mutation status (wild-type vs19DEL+L858R), and then discriminated between EGFR 19DEL and L858R (19DEL vs L858R), with AUCs of 0.76, 0.70, and 0.66, respectively (20). Song et al. employed DL to predict the mutation statuses of the EGFR (wild-type vs.19DEL+L858R), 19DEL (19Del vs. wild-type+L858R), and L858R (L858R vs. wild-type+19Del) with the AUC value 0.79 and 0.62, respectively (8). However, it is important to note that patients with EGFR Ex19del and L858R mutations exhibit significant differences in treatment response and prognosis (21). Radiomics quantifies medical images into multiple features and correlates them with gene characteristics (22). In contrast, Convolutional Neural Networks (CNN) evaluate image features at different levels. Deep Learning (DL) has advantages over radiomics as it learns complex features without manual delineation, can perform end-to-end tasks, and optimizes the loss function for better classification. DL outperforms radiomics in predicting EGFR mutations in lung cancer and has advantages in gene prediction for other cancers (23). In our study, we have developed a hybrid system that combines deep learning models with radiomics features. This strategy harnesses the pattern recognition capabilities of deep learning and the interpretability of radiomics features obtained through feature engineering. Our model has demonstrated superior performance, with higher AUC compared to only use machine learning or DL models. This approach showcases the synergy of combining these two techniques, resulting in improved results.

However, it is important to acknowledge the limitations of our study. Firstly, the generalizability of our findings may be limited as all patients were from the same center. Future studies should include data from multiple centers and diverse ethnicities to validate the results. Secondly, our study focused on NSCLC patients with non-small lung cancer, and the results may not be applicable to other histological subtypes. Finally, the radiomics-based approach requires precise labeling of tumor boundaries and processing of raw data, which can be time-consuming.

In future studies, it would be beneficial to collect data from multiethnic patient populations and multiple centers to enhance the generalizability of the findings. Additionally, an end-to-end approach that includes automatic tumor recognition, localization, and EGFR mutation prediction can be developed. Integrating radiomics features into deep learning models, along with clinical features and multi-level features, can further improve prediction performance. The resulting models can aid in determining appropriate EGFR-TKI therapy options for NSCLC patients in a non-invasive, reproducible, and cost-effective manner.

## Data Availability

The original contributions presented in the study are included in the article/**Supplementary Material**. Further inquiries can be directed to the corresponding author.
