# Supplementary material for: Advancing EGFR mutation subtypes prediction in NSCLC by combining 3D pretrained ConvNeXt, radiomics, and clinical features
Source: Front Oncol. 2024 Nov 15;14:1464555. doi: 10.3389/fonc.2024.1464555 (PMC11604581; doi:10.3389/fonc.2024.1464555)
Supplement: Supplementary file 1 [file SupplementaryFile1.docx]

**Supplementary**

**Supplementary Methods**

Imaging features that showed significant differences between the EGFR + and EGFR- include original_shape_Compactness1, original_shape_Compactness2, original_shape_Sphericity,original_shape_SphericalDisproportion,log-sigma-3-mm-3D_glszm_GrayLevelVariance,wavelet-LHL_glcm_InverseVariance,wavelet-HLH_glszm_ZonePercentage,wavelet-HHH_gldm_DependenceEntropy,wavelet-HHH_gldm_DependenceVariance,log-sigma-3-mm-3D_glrlm_RunEntropy,wavelet-HHH_gldm_DependenceNonUniformityNormalized.

Imaging features that showed significant differences between EGFR ex19del mutation and L858R include wavelet-LLH_firstorder_Kurtosis, wavelet-LHL_glcm_InverseVariance, wavelet-HHH_gldm_DependenceVariance, wavelet-LLL_gldm_DependenceEntropy, wavelet-LHH_glcm_ClusterTendency. These features may provide insights into the specific characteristics of EGFR ex19del mutation and L858R can potentially contribute to the understanding and prediction of mutation type.
