# Supplementary material for: Advancing EGFR mutation subtypes prediction in NSCLC by combining 3D pretrained ConvNeXt, radiomics, and clinical features
Source: Front Oncol. 2024 Nov 15;14:1464555. doi: 10.3389/fonc.2024.1464555 (PMC11604581; doi:10.3389/fonc.2024.1464555)
Supplement: Supplementary file 2 [file Table1.docx]

**Table S1 Clinical characteristics of patients with EGFR (+) and EGFR (-)**

| Clinical features | EGFR (+) | EGFR (-) | P-value^b^ |
| --- | --- | --- | --- |
|  | (n=351) | (n=381） |  |
| years^a^ | 59±11 | 57±12 | 0.204 |
| Gene |  |  | ＜0.001* |
| Male | 142(41) | 253(66) |  |
| Female | 209(59) | 65(34) |  |
| Smoking status  Smoking  non-smoking | 78(22)  273(78) | 171(74)  210(19) | ＜0.001* |
| T stage  T1  T2  T3  T4 | 205（58）  109（31）  27（7）  10（3） | 177(46)  128(54)  44（12）  31（8） | ＜0.001* |
| Lesion size（cm） | 2.8（2.0-3.4） | 3.0（2.0-4.6） | 0.002* |
