# Supplementary material for: Advancing EGFR mutation subtypes prediction in NSCLC by combining 3D pretrained ConvNeXt, radiomics, and clinical features
Source: Front Oncol. 2024 Nov 15;14:1464555. doi: 10.3389/fonc.2024.1464555 (PMC11604581; doi:10.3389/fonc.2024.1464555)
Supplement: Supplementary file 3 [file Table2.docx]

**Table S2 Clinical characteristics of patients with ex19del and L858R mutation**

| Clinical features | ex19del | L858R | P-value^b^ |
| --- | --- | --- | --- |
|  | (n=195) | (n=156） |  |
| years^a^ | 58±11 | 60±11 | 0.014* |
| Gene |  |  | 0.501 |
| Male | 82(42) | 60(39) |  |
| Female | 113(58) | 96(61) |  |
| Somking status  smoking  Non-smoking | 48(25)  147(75) | 30(19)  126(81) | ＜0.230 |
| T stage  T1  T2  T3  T4 | 117（60）  61（31）  10（5）  7（4） | 87(55)  48(31)  18（12）  3（2） | 0.389 |
| Lesion size（cm） | 2.7（1.9-3.3） | 2.9（2.0-3.8） | 0.064 |
